# Associations of active and passive tobacco exposure with elevated blood pressure in Korean adolescents

**DOI:** 10.4178/epih.e2024028

**Published:** 2024-02-13

**Authors:** Hyerin Park, Hyunsuk Jeong, Hyeon Woo Yim, Sanghyuk Bae

**Affiliations:** 1 Graduate School of Public Health and Healthcare Management, The Catholic University of Korea, Seoul, Korea; 2 Department of Preventive Medicine, College of Medicine, The Catholic University of Korea, Seoul, Korea

**Keywords:** Hypertension, Blood pressure, Smoking, Passive tobacco exposure

## Abstract

**OBJECTIVES:**

To test the hypothesis that tobacco exposure is associated with elevated blood pressure (EBP) in Korean adolescents, and that the association is dose dependent.

**METHODS:**

This cross-sectional study used data from the 2011-2020 Korea National Health and Nutrition Survey (KNHANES). Subjects were eligible if they were 13-18 years at the time of participation in KNHANES. Tobacco exposure was defined by urine cotinine level. The main outcomes were EBP and hypertension. Statistical analyses were conducted using SAS version 9.4 with appropriate sampling weights to account for the complex survey design, stratification, and cluster variable.

**RESULTS:**

A total of 2,518 adolescents was included in the analysis, representing 2.5 million Korean adolescents. The mean± standard deviation participant age was 15.3±1.7 years, and 55.3% were male. The number of participants with active tobacco smoke exposure was 283 (11.2%), passive tobacco smoke exposure was 145 (5.8%), and no smoke exposure was 2,090 (83.0%). Analysis of the 2,518 urine-cotinine-verified participants showed that tobacco smoke exposure had a significant effect on EBP: with an odds of elevated blood pressure of 3.00 (95% confidence interval [CI], 1.14 to 7.89). The odds of hypertension were 3.61 (95% CI, 1.13 to 11.49) in the active smoking group compared with the no tobacco exposure group after adjustment for potential confounders.

**CONCLUSIONS:**

It is necessary to present a range of public health plans to reduce tobacco exposure that affects adolescents’ blood pressure, and further research with a larger number of participants using urine cotinine as a biomarker is needed.

## GRAPHICAL ABSTRACT

[Fig f2-epih-46-e2024028]

## Key Message

Hypertension during adolescence is a leading cause of disease in adults. The relationship between smoking and hypertension has been studied, but findings between studies are conflicting. Nicotine is a known toxin, but the relationship between active and passive smoking and blood pressure in adolescents is not clear. So that we tested and found adolescents in Korea who were active smokers showed over 3-fold increased risk of elevated blood pressure.

## INTRODUCTION

Hypertension is a chronic disease that highly impacts health and health care costs [1]. Awareness, treatment, and control of hypertension have been increasing due to multidisciplinary public health programs and policies, but disease prevalence has not decreased [2]. Hypertension in adults is affiliated with numerous and serious sequelae, including stroke and cardiovascular and kidney diseases. Although hypertension in adolescents is not directly related to cardiovascular disease and mortality, many studies have shown that hypertension in children and adolescents is associated with adult hypertension [3].

Prevalence of hypertension in children and adolescents is around 1-5%, of which about 80% is the result of secondary conditions such as family history, body mass index (BMI), nutrition, and socioeconomic status [4]. Several studies have shown that smoking is a risk factor for hypertension, and exposure to cigarette smoking has an adverse effect on health throughout the lifespan [5,6]. One study reported that smoking was not significantly related to hypertension in children and adolescents [4]. In contrast, a recent study of data from the U.S.’ National Health and Nutrition Examination Survey (NHANES) reported that tobacco exposure in children and adolescents was significantly related to increased blood pressure [7].

A cohort study of adults in Korea that reported that smoking, confirmed as urinary cotinine level, was associated with lower prevalence of hypertension in men but was not related to hypertension in the entire population or in women [8,9]. However, no study has evaluated the relationship between cigarette-smoke exposure and blood pressure. This study evaluated the association between cigarette smoke exposure and increasing blood pressure and high blood pressure risk in Korean adolescents using data from the Korea National Health and Nutrition Survey (KNHANES).

## MATERIALS AND METHODS

### Study population

**Figure 1.**
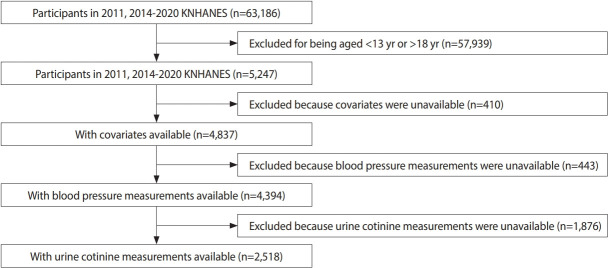
Flowchart of the process of participant selection and exclusion. KNHANES, Korea National Health and Nutrition Survey.

In this secondary-analysis study, we used data on KNHANES from 2011-2020 excluding years 2012 and 2013 as urinary cotinine was not measured. All survey protocols were approved by the Korea Disease Control and Prevention Agency (KDCA). Participants were included if they were 13-18 years old and had available blood pressure and urinary cotinine data. Of the 63,186 participants in the 2011-2020 KNHANES, 5,247 met the age criterion, but 410 were missing covariate data, 443 did not have blood pressure measurements, and 1,876 did not have urine cotinine measurements (Figure 1). Ultimately, 2,518 participants were included in the analyses.

### Main exposure variable

Tobacco exposure was divided into active and passive categories. Active smoking was defined as a urine cotinine level greater than or equal to 100 ng/mL, and passive smoking was defined as a urine cotinine level greater than or equal to 5 ng/mL and less than 100 ng/mL [10].

Urine cotinine level was measured by gas chromatography mass spectrometry using a Perkin Elmer Clarus 600T (Perkin Elmer, Turku, Finland) and high-performance liquid chromatography mass spectrometry using the Agilent 1100 Series with the API 4000 (AB Sciex, Redwood City, CA, USA). The lower limit of detection of this assay is 0.5 ng/mL [11].

### Main outcome variables

Elevated blood pressure (EBP) and hypertension were the main outcomes. All blood pressure measurements were performed manually by four nurses. Blood pressure was measured three times, and the average of the second and third measurements was used as the final value. EBP was defined as systolic/diastolic blood pressure greater than 120/80 mmHg, and hypertension was defined as systolic/diastolic blood pressure greater than 130/80 mmHg.

### Covariates

Variables that directly or indirectly affect smoking and blood pressure were identified from a literature review, and we used directed acyclic graph (DAG) analysis to identify minimal sufficient adjustment sets of confounders [12]. Age, sex, BMI, family smoking status, stress, and household income were included as confounding variables. The DAG results are presented in Supplementary Material 1. BMI was categorized into three groups-normal weight, overweight and obese-using the Pediatric and Adolescent Growth Chart 2017 [13]. Self-reported family smoking status was grouped as none, smoking, and not answered. Stress was divided into three categories–high (a report of very much or much), medium (a report of little or hardly), and not answered. Household income was classified into quartiles of low, medium low, medium high, and high. Among the confounding variables, the presence of missing values exceeding 70% for physical activity, and alcohol consumption was excluded from the analysis. Missing values for father’s and mother’s education were categorized into a single category and included in the analysis.

### Statistical analysis

The differences in prevalence of EBP and hypertension were analyzed for adolescents with available urinary cotinine data. The socio-demographic characteristics of the study participants according to active and passive smoking exposure were presented as number (% weighted), and the statistical differences between the two groups were assessed using the Rao-Scott chi-square test. Additional socio-demographic characteristics of the study participants for the normal, EBP and hypertension group were presented in a comparable manner. The associations between EBP, hypertension prevalence, and active or passive smoking exposure were examined using PROC SURVEYLOGISTIC to estimate odds ratios (ORs) and 95% confidence intervals (CIs). All statistical analyses were performed using SAS version 9.4 (SAS Institute Inc., Cary, NC, USA), taking into account complex sampling design by including weights, stratification variables, and clustering variables. A p-value < 0.05 was considered statistically significant.

### Ethics statement

This study received exemption approval from Catholic University Songeui Institutional Review Board (No. MC23EASI0023).

## RESULTS

### Participant characteristics

A total of 2,518 adolescents aged 13-18 years was included in the analysis, representing 2.5 million Korean adolescents. Among them, 2,090 (83.0%) were non-smokers, 283 (11.2%) were active smokers, and 145 (5.8%) were passive smokers. Mean± standard deviation (SD) participant age was 15.3 ± 1.7 years, and 1,398 (55.3%) were males. The prevalence of EBP and hypertension for non-smokers was 4.8% and 3.1%, respectively; those for passive smokers were 4.1% and 3.3% and those for active smokers were 7.5% and 2.7% (Table 1). Active smokers were more likely than passive smokers to be older (mean± SD age, 16.7± 1.3 vs. 15.7±1.6 years), male (76.8 vs. 58.8%), have higher levels of stress (38.1 vs. 24.8%), and have no insurance (3.2 vs. 0.2%) or medical assistance (8.7 vs. 3.4%). The prevalence of family smoking was similar in the passive smoking group compared with the active smoking group (29.1 vs. 28.8%) (Table 1).

When the total group of adolescents was divided by blood pressure levels, 2,301 (91.4%) were normal, 133 (5.3%) had EBP and 84 (3.3%) had hypertension. Differences were found in all sociodemographic characteristics excluding urine cotinine and residential type between the different blood pressure levels (Table 2).

### Associations between tobacco exposure and abnormal blood pressure

The OR for EBP was 1.93 (95% CI, 0.86 to 4.32), and the OR for hypertension was 2.35 (95% CI, 0.88 to 6.27) for participants with tobacco exposure compared with no tobacco exposure after adjusting potential confounders, including age and sex. The exposure category (passive tobacco exposure vs. active smoking) showed association with EBP (OR, 3.00; 95% CI, 1.14 to 7.89) and hypertension (OR, 3.61; 95% CI, 1.13 to 11.49) in the active smoking group. The relationships with EBP (OR, 0.90; 95% CI, 0.29 to 2.85) and hypertension (OR, 1.11; 95% CI, 0.28 to 4.44) were not significant in the passive smoking group after adjusting for all confounding variables (Table 3). In addition to adjusting for age, we also controlled for survey year. This adjustment was made because individuals of the same age but born in different years may introduce a potential source of error in the results, as presented in Supplementary Material 2. The OR for EBP and hypertension in the male only group exhibited the same pattern as the total group but was not statistically significant (Supplementary Material 3).

The association between a 1-ng/mL increase in urine cotinine and hypertension is shown in Table 4. The results show that a 1-ng/mL increase in urine cotinine tended to associate an increase in hypertension risk for both active and passive smokers, though the impact was not statistically significant.

Sensitivity analysis was done with lower cut-off level of the urine cotinine for active smoking from the current 100 ng/mL to 50 ng/mL, as recommended by the Society for Research on Nicotine and Tobacco [14]. Following a sensitivity analysis, the active smoking group demonstrated congruent outcomes that OR 2.39 for EBP and 3.38 for the hypertension after adjusting potential confounders. The sensitivity analysis is presented as Supplementary Material 4.

## DISCUSSION

This study revealed that adolescents in Korea who were active smokers showed a 3.6-fold increased hypertension and a 3.0-fold increased risk of EBP compared with non-smokers. A recent study involving 8,520 participants from the U.S. NHANES reported a significant association between EBP and hypertension and active and passive exposure to tobacco smoke in children and adolescents, where the exposed group had a 1.31-times higher likelihood of disease compared with the non-exposed group [7]. Previous studies have also shown that tobacco smoke exposure has an impact on EBP in certain children [15,16]. Long-term smoking has been linked to development of arterial sclerosis in adults, and a significant and rapid increase in arterial stiffness immediately after smoking a single cigarette has been observed [17]. Studies have also reported that children exposed to tobacco smoke experience a deterioration in endothelial function relative to nonexposed children [18]. Based on animal experiments and clinical trials, it is widely recognized that smoking exerts its effects by primarily stimulating the sympathetic nervous system, including at the neuronal terminals. This mechanism is known to cause persistent increases in blood pressure and can lead to sudden arrhythmias [19].

In a study analyzing data from Korea Youth Risk Behavior Web-based Survey (KYRBWS) conducted between 2005 and 2013, active smoking rates were reported as 14.4% for males and 4.6% for females, while passive smoking rates were 29.5% for males and 32.0% for females [20]. In the present study analyzing data from 2011 to 2020 from KNHANES, active smoking rates increased to 19.4% for males and 7.3% for females, while passive smoking rates decreased to 6.9% for males and 6.0% for females. It is important to note that the KYRBWS relies on self-reported surveys to determine smoking status. Therefore, the reported rates of active smoking may be underestimated compared to the actual prevalence. Additionally, the decrease in passive smoking rates may be influenced by the nationwide implementation of smoking bans in facilities such as restaurants and PC rooms, starting from 2013 under the amendment of the National Health Promotion Act by the Ministry of Health and Welfare.

During smoking, nicotine is absorbed into the bloodstream through the lungs and rapidly reaches the brain. Approximately 70-80% of inhaled nicotine is converted to cotinine in the body, and 10-15% of cotinine is excreted in the urine, while the remainder is converted to 4-oxo-4 (3-pyridyl) butanoic acid (keto acid). About 85% of keto acid is further converted to 4-hydroxy-4-(3-pyridyl) butanoic acid (hydroxy acid) and excreted in the urine [21]. Cotinine is the primary metabolite of nicotine and is considered a reliable biomarker for assessing smoking exposure. Nicotine can increase blood pressure through various biological mechanisms, including mimicking the actions of the sympathetic nervous system, regulating the renin-angiotensin system, and upregulating arginine, vasopressin, and endothelin-1 [22-24].

The association between tobacco exposure and EBP in children and adolescents has significant implications for public health. Notably, New Zealand has taken proactive measures to address smoking-related issues. In December 2022, legislation was passed, set to take effect in 2023, prohibiting the purchase of tobacco products by people born after January 1, 2009, throughout their lifetime. The objective of this legislation is to bring about a transformation in the smoking environment by restricting access to tobacco and reducing the number of tobacco retailers. These efforts aim to create an environment that discourages smoking and promotes better health outcomes among the population [25]. According to a press release from the KDCA in 2022, smoking-related deaths in 2019 in Korea numbered more than 58,000 people. Additionally, the estimated socioeconomic cost resulting from smoking was calculated as more than 12 trillion Korean won [26]. Furthermore, in 2019, Korea ranked fifth among the 36 Organization for Economic Cooperation and Development member countries for daily smoking rates among males aged 15 years and older [27]. Blood pressure during adolescence is associated with adult blood pressure, and EBP in adulthood is a recognized risk factor for cardiovascular diseases. Consequently, the impact of tobacco smoke exposure on elevation of blood pressure during adolescence is a significant focus in public health campaigns. Understanding and addressing this issue can contribute to preventive efforts aimed at reducing the burden of cardiovascular diseases in the population [7].

Potential limitations of this study are that, according to domestic research, cotinine may not be detectable within three days of smoking cessation [28]. People who have abstained from smoking for more than a week may be classified as non-smokers, despite having a history of active smoking. This study adopted the categorization of cotinine levels based on existing literature, defining active smoking as >100 ng/mL, passive smoking as 5-100 ng/mL, and non-smoking as < 5 ng/mL [10]. However, other studies have utilized a different threshold, defining active smoking as ≥ 50 ng/mL [29]. This variation in classification thresholds introduces the potential for misclassification errors, and there is a possibility of including a significant number of passive smokers among the self-reported active smokers. Last, this is a cross-sectional that cannot determine causal relationships.

However, this study utilized data from the nationally representative KNHANES, enabling a large-scale examination of the relationship between tobacco smoke exposure and blood pressure in adolescents. This study presents evidence that smoking, specifically active smoking, may increase the risk of EBP in adolescents. Therefore, this study offers valuable insights for prevention of smoking during adolescence. In future studies, it would be beneficial to conduct additional research targeting a larger number of adolescents using urine cotinine. This would provide more comprehensive insights into the relationship between tobacco smoke exposure and blood pressure among adolescents.

## Figures and Tables

**Figure f2-epih-46-e2024028:**
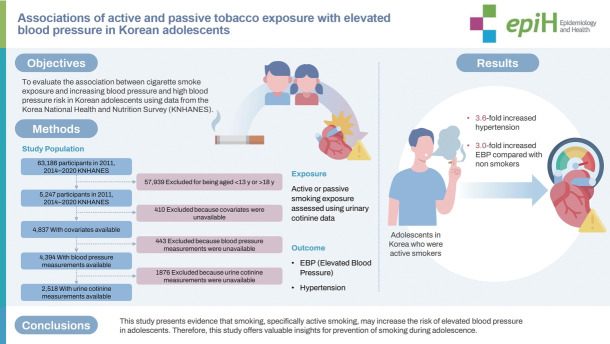


**Table 1.** Socio-demographic characteristics of study participants aged 13-18 years from 2011-2020 Korea National Health and Nutrition Examination Surveys (n=2,518)

Characteristics	All (n=2,518)	Tobacco smoke exposure			p-value
		None (n=2,090)	Passive (n=145)	Active (n=283)	
Age (yr)	15.3±1.7	15.1±1.7	15.7±1.6	16.7±1.3	<0.001
Sex					
Male	1,398 (55.3)	1,092 (51.2)	87 (58.8)	219 (76.8)	<0.001^1^
Female	1,120 (44.7)	998 (48.8)	58 (41.2)	64 (23.2)	
Body mass index (kg/m^2^)	21.6±3.9	21.5±3.9	21.7±3.8	21.9±3.9	<0.001
Normal	1,957 (78.2)	1,621 (78.7)	106 (71.6)	230 (78.7)	<0.001^1^
Overweight	239 (8.5)	205 (8.9)	17 (13.0)	17 (8.9)	
Obese	322 (13.3)	264 (12.4)	22 (15.4)	36 (12.4)	
Family smoking					
None	1,465 (56.3)	1,272 (59.8)	68 (41.7)	125 (43.1)	<0.001^1^
Smoking	518 (21.5)	395 (19.6)	39 (29.1)	84 (28.8)	
Not answered	535 (22.2)	423 (20.6)	38 (29.2)	74 (28.1)	
Stress					
High	704 (28.5)	565 (27.1)	35 (24.8)	104 (38.1)	<0.001^2^
Medium	1,802 (71.0)	1,517 (72.5)	109 (74.8)	176 (60.8)	
Not answered	12 (0.5)	8 (0.4)	1 (0.4)	3 (1.1)	
Economic status					
Low	259 (11.4)	195 (10.2)	16 (12.4)	48 (17.7)	<0.001^1^
Medium low	601 (24.8)	484 (24.3)	43 (31.7)	74 (24.9)	
Medium high	875 (33.9)	750 (34.9)	41 (28.4)	84 (30.8)	
High	783 (29.8)	661 (30.6)	45 (27.6)	77 (26.6)	
Insurance					
Yes	2,390 (94.0)	2,001 (95.2)	134 (96.4)	255 (88.1)	0.008^2^
No	23 (1.1)	13 (0.6)	2 (0.2)	8 (3.2)	
Medical care	105 (4.9)	76 (4.2)	9 (3.4)	20 (8.7)	
Urine cotinine, median [IQR] (ng/mL)	0.9 [0.4-2.5]	0.7 [0.4-1.3]	8.3 [6.3-15.8] 786.5	[427.7-1,052.6]	<0.001
Residence type					
General	914 (41.4)	716 (38.1)	73 (59.5)	125 (51.8)	<0.001^1^
Apartment	1,604 (58.6)	1,374 (61.9)	72 (40.5)	158 (48.2)	
Residence area					
Urban	2,156 (85.5)	1,811 (86.5)	116 (76.0)	229 (83.8)	<0.001^1^
Rural	362 (14.5)	279 (13.5)	29 (24.0)	54 (16.2)	
Education					
Father					
Elementary school	37 (1.8)	25 (1.6)	3 (1.3)	9 (3.1)	<0.001^1^
Middle school	138 (6.0)	105 (5.1)	9 (5.5)	24 (11.6)	
High school	645 (25.8)	525 (25.6)	39 (23.7)	81 (27.8)	
College	878 (32.7)	787 (36.1)	34 (22.0)	57 (18.6)	
Not answered	820 (33.7)	648 (31.6)	60 (47.5)	112 (38.9)	
Mother					
Elementary school	39 (1.8)	21 (1.1)	5 (2.8)	13 (5.6)	<0.001^1^
Middle school	97 (4.3)	71 (3.9)	6 (4.4)	20 (7.0)	
High school	1,072 (42.1)	885 (42.1)	66 (46.5)	121 (39.3)	
College	955 (36.6)	853 (40.2)	37 (23.7)	65 (22.4)	
Not answered	355 (15.2)	260 (12.7)	31 (22.6)	64 (25.7)	
Hypertension					
Father					
No	1,740 (72.1)	1,426 (71.6)	113 (76.6)	201 (72.8)	<0.001^1^
Yes	306 (12.4)	250 (12.4)	18 (13.5)	38 (12.1)	
Not answered	472 (15.5)	414 (16.0)	14 (9.9)	44 (15.1)	
Mother					
No	1,926 (79.8)	1,584 (79.6)	122 (82.5)	220 (79.8)	<0.001^1^
Yes	118 (4.6)	92 (4.3)	9 (7.6)	17 (4.8)	
Not answered	474 (15.6)	414 (16.1)	14 (9.9)	46 (15.4)	
Elevated BP^3^	133 (5.1)	104 (4.8)	6 (4.1)	23 (7.5)	<0.001^1^
Hypertension^4^	84 (3.1)	71 (3.1)	2 (3.3)	11 (2.7)	<0.001^1^

Values are presented as mean±standard deviation or number (weighted %). BP, blood pressure; IQR, interquartile range; SBP, systolic blood pressure; DBP, diastolic blood pressure.

1 Chi-square test.

2 Fisher’s exact test.

3 SBP/DBP is greater than 120/80 mmHg.

4 SBP/DBP is greater than 130/80 mmHg.

**Table 2.** Socio-demographic characteristics of study participants aged 13-18 years from 2011-2020 Korea National Health and Nutrition Examination Surveys-group divided by levels of BP (n=2,518)

Characteristics	Levels of BP			p-value
	Normal (n=2,301)	Elevated BP (n=133)^1^	Hypertension (n=84)^2^	
Age (yr)	15.3±1.7	15.6±1.7	15.8±1.6	<0.001
Sex				
Male	1,241 (53.9)	83 (60.5)	74 (88.6)	<0.001^3^
Female	1,060 (46.1)	50 (39.5)	10 (11.4)	
Body mass index (kg/m^2^)	21.3±3.6	24.1±4.8	25.8±5.3	<0.001
Normal	1,841 (80.6)	86 (63.8)	30 (30.7)	<0.001^3^
Overweight	219 (8.5)	6 (4.1)	14 (16.0)	
Obese	241 (10.9)	41 (32.1)	40 (53.3)	
Family smoking				
None	1,343 (56.6)	82 (58.0)	40 (44.3)	<0.001^3^
Smoking	470 (21.7)	23 (13.9)	25 (29.7)	
Not answered	488 (21.7)	28 (28.1)	19 (26.1)	
Stress				
High	667 (29.2)	26 (21.1)	11 (19.7)	<0.001^4^
Medium	1,623 (70.3)	106 (78.3)	73 (80.3)	
Not answered	11 (0.5)	1 (0.5)	0 (0.3)	
Economic status				
Low	241 (11.6)	15 (10.3)	3 (7.0)	<0.001^3^
Medium low	539 (24.4)	36 (29.3)	26 (31.4)	
Medium high	802 (34.1)	41 (29.1)	32 (36.1)	
High	719 (30.0)	41 (31.4)	23 (25.4)	
Insurance				
Yes	2,178 (93.6)	130 (98.0)	82 (97.0)	<0.001^4^
No	22 (1.1)	1 (1.3)	0 (0.0)	
Medical care	101 (5.3)	2 (0.7)	2 (3.0)	
Urine cotinine, median [IQR] (ng/mL)	0.9 [0.4-2.3]	1.2 [0.5-3.9]	0.8 [0.5-2.7]	0.277
Residence type				
General	819 (40.8)	59 (41.4)	36 (38.1)	0.080^3^
Apartment	1,482 (59.2)	74 (58.6)	48 (61.9)	
Residence area				
Urban	1,970 (86.5)	107 (82.0)	79 (93.8)	<0.001^3^
Rural	331 (13.5)	26 (18.0)	5 (6.2)	
Education				
Father				
Elementary school	37 (2.0)	0 (0.0)	0 (0.0)	<0.001^3^
Middle school	122 (6.0)	5 (4.1)	11 (11.9)	
High school	587 (25.9)	38 (24.5)	20 (23.8)	
College	805 (32.7)	49 (36.5)	24 (25.6)	
Not answered	750 (33.4)	41 (34.8)	29 (38.6)	
Mother				
Elementary school	36 (1.9)	0 (0.0)	3 (3.7)	<0.001^3^
Middle school	86 (4.2)	10 (8.4)	1 (1.1)	
High school	952 (41.1)	67 (48.8)	53 (61.3)	
College	901 (37.8)	36 (25.9)	18 (19.1)	
Not answered	326 (15.1)	20 (16.9)	9 (14.8)	
Hypertension				
Father				
No	1,601 (69.6)	90 (70.0)	49 (53.9)	<0.001^3^
Yes	270 (11.7)	16 (14.7)	20 (27.0)	
Not answered	430 (18.7)	27 (15.4)	15 (19.2)	
Mother				
No	1,769 (80.3)	91 (73.5)	66 (79.6)	<0.001^3^
Yes	103 (4.3)	13 (9.8)	2 (3.5)	
Not answered	429 (15.4)	29 (16.8)	16 (19.9)	

BP, blood pressure; IQR, interquartile range; SBP, systolic blood pressure; DBP, diastolic blood pressure.

1 SBP/DBP is greater than 120/80 mmHg.

2 SBP/DBP is greater than 130/80 mmHg.

3 Chi-square test.

4 Fisher’s exact test.

**Table 3.** Associations of tobacco exposure with abnormal blood pressure in 2,518 participants aged 13-18 years from the 2011-2020 Korea National Health and Nutrition Examination Surveys^1^

Exposure status^2^	Elevated blood pressure (n=133)^3^			Hypertension (n=84)^4^		
	Model 1	Model 2	Model 3	Model 1	Model 2	Model 3
No tobacco exposure (n=2,090)	1.00 (reference)	1.00 (reference)	1.00 (reference)	1.00 (reference)	1.00 (reference)	1.00 (reference)
Any tobacco exposure (n=428)^5^	1.07 (0.50, 2.28)	1.66 (0.75, 3.65)	1.93 (0.86, 4.32)	1.48 (0.59, 3.71)	2.16 (0.83, 5.63)	2.35 (0.88, 6.27)
Passive tobacco exposure (n=145)	0.75 (0.22, 2.55)	0.91 (0.26, 3.17)	0.90 (0.29, 2.85)	0.99 (0.24, 4.07)	1.17 (0.28, 4.92)	1.11 (0.28, 4.44)
Active smoking (n=283)	1.34 (0.57, 3.18)	2.32 (0.94, 5.70)	3.00 (1.14, 7.89)	1.89 (0.65, 5.51)	3.03 (1.00, 9.15)	3.61 (1.13, 11.49)

SBP, systolic blood pressure; DBP, diastolic blood pressure.

1 Model 1: Unadjusted; Model 2: Adjusted for age and sex; Model 3: Adjusted for age, sex, body mass index, economic status, family smoking status, stress, and family history of hypertension (paternal or maternal).

2 No tobacco exposure was defined as a urine cotinine level below 5 ng/mL; Passive tobacco exposure was defined as a urine cotinine level of at least 5 ng/mL and less than 100 ng/mL; Active smoking was defined as a urine cotinine level of at least 100 ng/mL.

3 Defined as SBP/DBP greater than 120/80 mmHg.

4 Defined as SBP/DBP greater than 130/80 mmHg.

5 The reference category was no tobacco exposure.

**Table 4.** Association between 1-ng/mL increase in urine cotinine and prevalence of hypertension^1^

Exposure status^2^	Model 1	Model 2	Model 3
No tobacco exposure (n=2,090)	1.0000 (reference)	1.0000 (reference)	1.0000 (reference)
Passive tobacco exposure (n=145)	1.0002 (0.9994, 1.0011)	1.0007 (0.9997, 1.0018)	1.0009 (0.9996, 1.0022)
Active smoking (n=283)	1.0004 (0.9994, 1.0014)	1.0008 (0.9997, 1.0020)	1.0009 (0.9996, 1.0023)

1 Model 1: Unadjusted; Model 2: Adjusted for age and sex; Model 3: Adjusted for age, sex, body mass index, economic status, family smoking, stress, family history of hypertension-father and mother.

2 No tobacco exposure is defined as urine cotinine level is below 5 ng/mL; Passive tobacco exposure is defined as urine cotinine is greater than or equal to 5 ng/mL and less than 100 ng/mL; Active smoking is defined as urine cotinine is greater than or equal to 100 ng/mL.

## Data Availability

The original data for this study are freely and publicly available from the Korea Disease Control and Prevention Agency (https://knhanes.kdca.go.kr/knhanes/sub03/sub03_02_05.do).

## Supplementary materials

Supplementary Material 1. Directed Acyclic Graph (DAG) to identify minimally sufficient adjustment sets of confounders

Supplementary Material 2. Association of tobacco exposure with abnormal blood pressure in 2,518 participants aged 13–18 years from 2011-2020 Korea National Health and Nutrition Examination Surveys controlled for survey year

Supplementary Material 3. Association of tobacco exposure with abnormal blood pressure in 1398 male participants aged 13–18 years from 2011-2020 Korea National Health and Nutrition Examination Surveys

Supplementary Material 4. Sensitivity analysis with lower cut-off level of the urine cotinine for active smoking (50 ng/mL) in the association of tobacco exposure with abnormal blood pressure in 2,518 participants aged 13–18 years from 2011-2020 Korea National Health and Nutrition Examination Surveys
