# Supplementary material for: Associations of active and passive tobacco exposure with elevated blood pressure in Korean adolescents
Source: Epidemiol Health. 2024 Feb 13;46:e2024028. doi: 10.4178/epih.e2024028 (PMC11040219; doi:10.4178/epih.e2024028)

Supplementary Material 1. Directed Acyclic Graph (DAG) to identify minimally sufficient adjustment sets of confounders


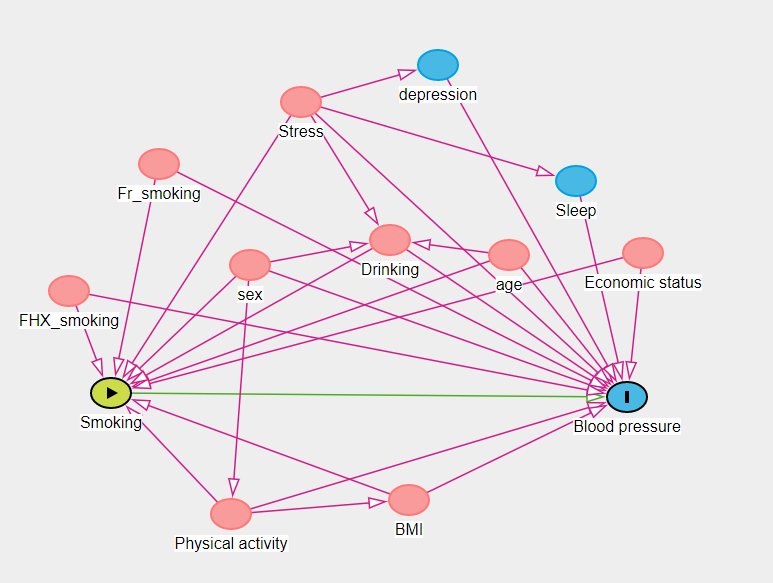

Supplement: Supplementary Material 1. — Directed Acyclic Graph (DAG) to identify minimally sufficient adjustment sets of confounders [file epih-46-e2024028-Supplementary-1.docx]
