# Supplementary material for: Associations of active and passive tobacco exposure with elevated blood pressure in Korean adolescents
Source: Epidemiol Health. 2024 Feb 13;46:e2024028. doi: 10.4178/epih.e2024028 (PMC11040219; doi:10.4178/epih.e2024028)
Supplement: Supplementary Material 2. — Association of tobacco exposure with abnormal blood pressure in 2,518 participants aged 13–18 years from 2011-2020 Korea National Health and Nutrition Examination Surveys controlled for survey year [file epih-46-e2024028-Supplementary-2.docx]

Supplementary Material 2. Association of tobacco exposure with abnormal blood pressure in 2,518 participants aged 13–18 years from 2011-2020 Korea National Health and Nutrition Examination Surveys controlled for survey year

|  | Elevated Blood Pressure (n=133) ^a^ | | |  | Hypertension (n=84) ^b^ | | |
| --- | --- | --- | --- | --- | --- | --- | --- |
| Exposure Status ^f^ | Model 1^c^ | Model 2^d^ | Model 3^e^ |  | Model 1^c^ | Model 2^d^ | Model 3^e^ |
| No Tobacco Exposure  (n=2090) | 1 (ref) | 1 (ref) | 1 (ref) |  | 1 (ref) | 1 (ref) | 1 (ref) |
| Passive Tobacco Exposure (n=145)  Active Smoking  (n=283) | 0.75  (0.22-2.55)  1.34  (0.57-3.18) | 0.79  (0.23-2.75)  2.31  (0.94-5.70) | 0.79  (0.25-2.49)  2.89  (1.09-7.71) |  | 0.99  (0.24-4.07)  1.89  (0.65-5.51) | 1.17  (0.26-5.33)  3.06  (1.01-9.29) | 1.16  (0.27-5.00)  3.62  (1.12-11.70) |

^a^ SBP/DBP is defined as greater than 120/80mmHg.

^b^ SBP/DBP is defined as greater than 130/80mmHg.

^c^ unadjusted

^d^ Adjusted for age, sex, and survey year

^e^ Adjusted for age, sex, BMI, economic status, family smoking, stress, family history of hypertension-father and mother, survey year

^f^ No tobacco exposure is defined as urine cotinine level is below 5ng/ml, Passive Tobacco exposure is defined as urine cotinine is greater than or equal to 5ng/ml and less than 100ng/ml, Active Smoking is defined as urine cotinine is greater than or equal to 100ng/ml
