# Supplementary material for: Associations of active and passive tobacco exposure with elevated blood pressure in Korean adolescents
Source: Epidemiol Health. 2024 Feb 13;46:e2024028. doi: 10.4178/epih.e2024028 (PMC11040219; doi:10.4178/epih.e2024028)
Supplement: Supplementary Material 3. — Association of tobacco exposure with abnormal blood pressure in 1398 male participants aged 13–18 years from 2011-2020 Korea National Health and Nutrition Examination Surveys [file epih-46-e2024028-Supplementary-3.docx]

Supplementary Material 3. Association of tobacco exposure with abnormal blood pressure in 1398 male participants aged 13–18 years from 2011-2020 Korea National Health and Nutrition Examination Surveys

|  | Elevated Blood Pressure (n=83) ^a^ | | |  | Hypertension (n=74) ^b^ | | |
| --- | --- | --- | --- | --- | --- | --- | --- |
| Exposure Status ^f^ | Model 1^c^ | Model 2^d^ | Model 3^e^ |  | Model 1^c^ | Model 2^d^ | Model 3^e^ |
| No Tobacco Exposure  (n=1092) | 1 (ref) | 1 (ref) | 1 (ref) |  | 1 (ref) | 1 (ref) | 1 (ref) |
| Passive Tobacco Exposure (n=87)  Active Smoking  (n=219) | 0.73  (0.20-2.71)  1.73  (0.72-4.14) | 0.79  (0.21-2.96)  2.11  (0.85-5.25) | 0.83  (0.25-2.75)  2.58  (0.95-6.70) |  | 0.86  (0.19-3.95)  2.90  (0.96-8.72) | 0.85  (0.18-3.97)  2.81  (0.87-9.05) | 0.79  (0.17-3.71)  3.21  (0.93-11.05) |

^a^ SBP/DBP is defined as greater than 120/80mmHg.

^b^ SBP/DBP is defined as greater than 130/80mmHg.

^c^ unadjusted

^d^ Adjusted for age and sex

^e^ Adjusted for age, sex, BMI, economic status, family smoking, stress, family history of hypertension-father and mother.

^f^ No tobacco exposure is defined as urine cotinine level is below 5ng/ml, Passive Tobacco exposure is defined as urine cotinine is greater than or equal to 5ng/ml and less than 100ng/ml, Active Smoking is defined as urine cotinine is greater than or equal to 100ng/ml
