# Supplementary material for: Associations of active and passive tobacco exposure with elevated blood pressure in Korean adolescents
Source: Epidemiol Health. 2024 Feb 13;46:e2024028. doi: 10.4178/epih.e2024028 (PMC11040219; doi:10.4178/epih.e2024028)
Supplement: Supplementary Material 4. — Sensitivity analysis with lower cut-off level of the urine cotinine for active smoking (50 ng/mL) in the association of tobacco exposure with abnormal blood pressure in 2,518 participants aged 13–18 years from 2011-2020 Korea National Health and Nutrition Examination Surveys [file epih-46-e2024028-Supplementary-4.docx]

Supplementary Material 4. Sensitivity analysis with lower cut-off level of the urine cotinine for active smoking (50 ng/mL) in the association of tobacco exposure with abnormal blood pressure in 2,518 participants aged 13–18 years from 2011-2020 Korea National Health and Nutrition Examination Surveys

|  | Elevated Blood Pressure (n=133) ^a^ | | |  | Hypertension (n=84) ^b^ | | |
| --- | --- | --- | --- | --- | --- | --- | --- |
| Exposure Status ^f^ | Model 1^c^ | Model 2^d^ | Model 3^e^ |  | Model 1^c^ | Model 2^d^ | Model 3^e^ |
| No Tobacco Exposure  (n=2090)  Any Tobacco ^g^ Exposure  (n=428) | 1 (ref)  1.07  (0.50-2.28) | 1 (ref)  1.65  (0.75-3.65) | 1 (ref)  1.93  (0.86-4.32) |  | 1 (ref)  1.48  (0.59-3.71) | 1 (ref)  2.16  (0.83-5.63) | 1 (ref)  2.35  (0.88-6.27) |
| Passive Tobacco Exposure (n=128)  Active Smoking  (n=300) | 0.96  (0.21-4.50)  1.13  (0.52-2.45) | 1.17  (0.24-5.67)  1.90  (0.85-4.24) | 1.11  (0.27-4.60)  2.39  (1.00-5.72) |  | 0.81  (0.15-4.47)  1.81  (0.68-4.47) | 0.97  (0.17-5.57)  2.88  (1.06-7.84) | 0.88  (0.17-4.49)  3.38  (1.17-9.75) |

^a^ SBP/DBP is defined as greater than 120/80mmHg.

^b^ SBP/DBP is defined as greater than 130/80mmHg.

^c^ unadjusted

^d^ Adjusted for age and sex

^e^ Adjusted for age, sex, BMI, economic status, family smoking, stress, family history of hypertension-father and mother.

^f^ No tobacco exposure is defined as urine cotinine level is below 5ng/ml, Passive Tobacco exposure is defined as urine cotinine is more than or equal to 5ng/ml and less than 50ng/ml, Active Smoking is defined as urine cotinine is more than or equal to 50ng/ml

^g^ Reference category was no tobacco exposure.
